# The role of the unfolded protein response in arrhythmias

**DOI:** 10.1080/19336950.2018.1516985

**Published:** 2018-09-29

**Authors:** Man Liu, Samuel C Dudley

**Affiliations:** Division of Cardiology, Department of Medicine, The Lillehei Heart Institute, University of Minnesota at Twin Cities, Minneapolis, USA

**Keywords:** Cardiac ion channels, chaperones, early afterdepolarizations, ER stress, long QT, oxidative stress, RNA stability, sudden cardiac death

## Abstract

Human heart failure is characterized by arrhythmogenic electrical remodeling consisting mostly of ion channel downregulations. Reversing these downregulations is a logical approach to antiarrhythmic therapy, but understanding the pathophysiological mechanisms of the reduced currents is crucial for finding the proper treatments. The unfolded protein response (UPR) is activated by endoplasmic reticulum (ER) stress and has been found to play pivotal roles in different diseases including neurodegenerative diseases, diabetes mellitus, and heart disease. Recently, the UPR is reported to regulate multiple cardiac ion channels, contributing to arrhythmias in heart disease. In this review, we will discuss which UPR modulators and effectors could be involved in regulation of cardiac ion channels in heart disease, and how the understanding of these regulating mechanisms may lead to new antiarrhythmic therapeutics that lack the proarrhythmic risk of current ion channel blocking therapies.

## Introduction

### Heart failure and arrhythmias

The foundation of a regular heart rhythm is the cardiac action potential (AP) of cardiomyocyte. The cardiac AP relies upon highly regulated active and passive ion transport through Na^+^, K^+^, Ca^2+^ and other channels and transporters [1]. The cardiac Na^+^ channel (Na_v_1.5) governs phase 0 of depolarization of the AP. The K^+^ and Ca^2+^ channels determine the characteristic plateau of phase 2. K^+^ channels are also responsible for the phases 3 and 4 (the resting membrane potential) of the AP. Ca^2+^ channels and the Na^+^/Ca^2+^ exchanger (NCX) contribute to excitation-contraction coupling and pacemaker activities. Any disturbance of these ion channels can alter the delicate balance between depolarizing and repolarizing ionic currents, leading to slow conduction and prolongation of the AP duration (APD) that are observed in human heart failure.

Human heart failure is associated with life-threatening arrhythmias. Understanding the pathophysiological mechanisms of arrhythmias is crucial for discovering safe and efficacious new therapies. Early studies show that arrhythmias occur by three fundamental mechanisms: enhanced automaticity, triggered arrhythmias, or reentry. In each case, the initiation of the arrhythmia is secondary to excess, undesirable electrical activity. Based on this concept, ion channel blocking drugs were developed to treat arrhythmias. While these drugs are partially effective, their efficacy is limited by proarrhythmic risk. In other words, these drugs can generate arrhythmias as well as prevent them. This paradox becomes more understandable if one realizes that in most cardiac pathological conditions, ion channels are downregulated already. Blocking these channels further may reduce undesirable electrical activity, but it also exacerbates the underlying electrical defect responsible for arrhythmia generation.

Changes of cardiac ion channels and transporters underlie the increased arrhythmic risk in cardiomyopathy. For many ion channels, heart failure results in downregulation of the channel current. For example, Na_v_1.5 protein and the macroscopic Na^+^ current (I_Na_) are reduced in human heart failure and contribute to a decreased upstroke velocity (dV/dt_max_) of the AP phase 0. These changes can jeopardize the impulse propagation and decrease conduction velocity in heart tissue, causing conduction disturbances and ventricular arrhythmias [2–8]. Animal model studies reveal reductions in K^+^ currents including the transient outward current (I_to_, conducted mainly by rapidly inactivating K^+^ channel K_v_4.3), inward rectifying current (I_K1_, conducted mainly by the inward rectifying K^+^ channel Kir2.1), and slow delayed rectifying current (I_Ks_, conducted by slowly inactivating K^+^ channel K_v_LQT1). These reductions are responsible for prolonged APD, afterdepolarizations, heterogeneous repolarization patterns and ventricular arrhythmias [9–15]. Decreased L-type Ca^2+^ current (I_CaL_, conducted by Ca_v_1.2) can cause repolarization delay and early and delayed afterdepolarizations (EADs and DADs). Nevertheless, the reasons for these changes remain unknown. Understanding these changes and their origins should allow for more effective therapeutic strategies free from the proarrhythmic effects, which limit the use of approved ion channel blocking drugs.

### The importance of the unfolded protein response in heart function

Adult cardiomyocytes lack significant regenerative potential and require a vital balance of its contents such as sarcomeres, membrane ion channel proteins, and mitochondria to maintain viability and function throughout the life of the individual. Therefore, protein quality control is crucial for cardiomyocyte survival and function. The unfolded protein response (UPR) is one important mechanism of protein quality control in the endoplasmic reticulum (ER) to monitor and regulate misfolded and unfolded proteins [16]. The sarco/endoplasmic reticulum (SER) of cardiomyocytes is not only the place where membrane proteins, such as cardiac ion channels undergo synthesis, folding, and assembling, but the SER serves also as a Ca^2+^ reservoir for a normal excitation-contractility coupling. Therefore, the SER of cardiomyocytes is critical not only for general cellular function but also for myocyte contractility.

Activated UPR can inhibit nascent protein synthesis to attenuate the ER stress, but this can also cause reduced expression of essential proteins, which will affect cell function and even induce cell apoptosis when the UPR activation is severe and prolonged [17,18]. The UPR has been investigated extensively in neurodegenerative diseases and diabetes but not in heart disease. In the past decade, the UPR has been found to play important roles in pathological cardiac hypertrophy [19,20], dilated cardiomyopathy [21], ischemic cardiomyopathy [22–24], diabetic heart disease [25,26], and human heart failure [6,27]. There are many excellent review articles discussing the ER stress and the heart [28], the biology of the ER stress in heart [29,30], the ER stress and ischemic heart disease [30,31], and targeting the ER stress and the UPR in cardiovascular disease [32]. Previously, we have reviewed the role of the UPR in heart disease and cardiac arrhythmias and discussed the rationale for and the challenges to target the UPR in heart disease for treatment of arrhythmias [33,34]. In this review, we will discuss which UPR modulators and effectors could be involved in regulation of cardiac ion channels in heart disease and how the understanding of these regulating mechanisms may help point out new directions for future therapeutics.

## What is the UPR?

### Three arms of the UPR

The UPR is highly conserved in evolution from yeast to all mammalians. It is initially an adaptive stress response and plays protective roles for cell survival by eliminating unfolded or misfolded proteins. Nevertheless, when prolonged ER stress occurs, the UPR can lead to cell apoptosis. When under ER stress, unfolded or misfolded protein accumulation triggers the dissociation of glucose regulated protein-78 (Grp78) from the three main UPR sensors, double stranded RNA-activated protein kinase-like ER kinase (PERK), inositol-requiring ER-to-nucleus signal kinase 1 (IRE1), and activating transcription factor-6α (ATF6α), leading to their activation.

**Figure 1.**
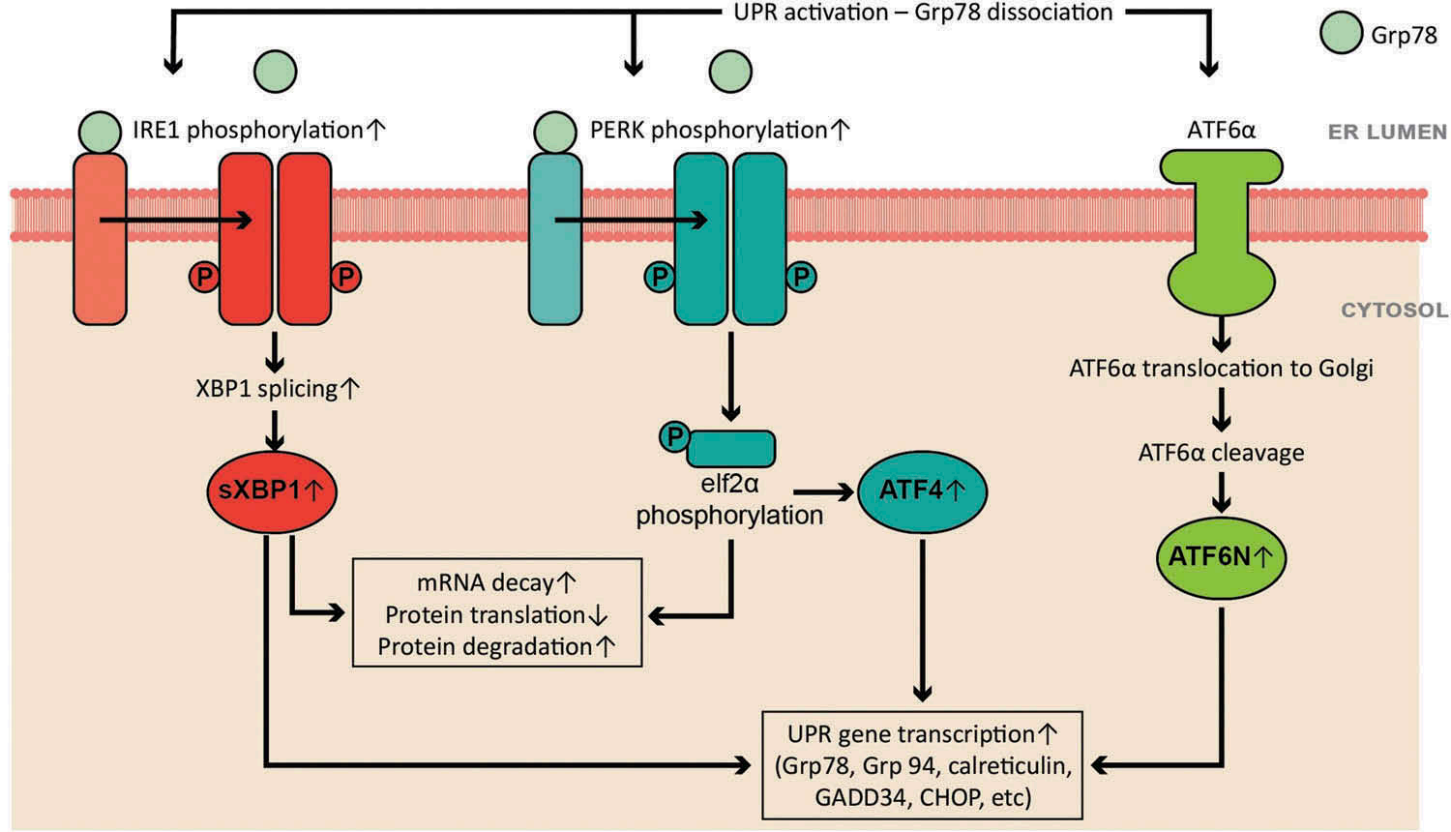
The scheme of the unfolded protein response (UPR) signaling cascades and functions.

Activation of the three UPR sensors initiates three complicated signal cascades (Figure 1) to increase protein folding capacity by increasing UPR genes expression and translation (such as ER chaperone proteins Grp78, Grp94, calreticulin, and GADD34) and to reduce ER protein burden by enhancing mRNA decay, inhibiting protein translation, and accelerating protein degradation. When Grp78 dissociates, PERK and IRE1 oligomerize, become phosphorylated, and induce activation of downstream effectors: phosphorylation of eukaryotic initiation factor 2α (eIF2α) and X-box binding protein 1 (XBP1) splicing, respectively. Phosphorylated eIF2α inhibits ribosomal-mRNA interactions, leading to subsequent mRNA degradation and nascent protein translation reduction. Phosphorylation of eIF2α also enhances gene expression of activating transcription factor 4 (ATF4), which increases the gene expression of ER chaperone proteins. Spliced XBP1 (sXBP1) degrades mRNA, upregulates gene expression of ER chaperones, and enhances ER-associated protein degradation. When Grp78 dissociates from ATF6α, it translocates to Golgi and is cleaved to the activated form of ATF6α, ATF6N (cleaved N-terminus of ATF6α). ATF6N translocates to the nucleus to enhance the gene expression of UPR targets such as ER chaperones. The signaling cascades of the three arms have been investigated mainly in neural degeneration diseases and diabetes mellitus [35–38], although a wide variety of cardiovascular diseases have been associated with the UPR activation, such as ischemia/reperfusion, myocardial infarction, hypertension, and heart failure [32–34,39–42].

### What is known about the UPR in heart disease?

In heart disease, the ER is often under stress from oxidative stress, hypoxia, and glucose deprivation. In the past two decades, activated UPR has been reported in ischemia/reperfusion [43–46], dilated cardiomyopathy [21], myocardial infarction [23,47–49], hypertension [50–53], diabetic cardiomyopathy [25,54,55], and heart failure [6,33,51,56–58] with increased expression of ER chaperones (Grp78 and calreticulin) and effectors from all three UPR arms. For example, the PERK and IRE1 arms are activated in human failing hearts with increased mRNA and/or protein levels of Grp78, sXBP1, PERK, ATF4, and CCAAT-enhancer-binding protein homologous protein (CHOP) [6,59]. Elevated Grp78 and CHOP are reported in mouse models of heart failure [51,60]. Our group found activated UPR in human failing heart tissue with elevated PERK, CHOP and calreticulin [6]. Elevated ATF6 protein levels occur in heart failure patients with dilated and ischemic cardiomyopathy [21].

When the ER homeostasis is disturbed, not only is the UPR is activated, Ca^2+^ homeostasis and protein posttranslational modifications can be altered as well. Activated UPR disturbs Ca^2+^ handling and increases oxidative stress and cell apoptosis, all of which have been reported to contribute to the pathogenesis of diabetic cardiomyopathy [23]. SER Ca^2+^ ATPase isoform 2a (SERCA2a) and 3f (SERCA3f) are both altered in heart failure. SERCA3f is upregulated in human failing hearts, and overexpression of this Ca^2+^ pump increases XBP1 splicing and Grp78 expression [61]. This indicates that SERCA3f is likely an UPR effector and participates in the ER stress in human heart failure. PERK helps to maintain SERCA2a levels in a transaortic constriction model of heart failure, a potentially salutary effect [53].

## The UPR and arrhythmias

Heart failure is characterized by arrhythmogenic electrical remodeling consisting mostly of ion channel downregulations. Activated UPR leads to suppression of nascent protein translation, which has been found to affect membrane expression of cardiac ion channels [6,62]. In human failing heart, abnormal splice variants of *SCN5A* encoding the α subunit of cardiac Na_v_1.5 are elevated and result in truncated, nonfunctional channel proteins trapped in the ER. This leads to PERK activation and causes downregulation of the full-length normal Na_v_1.5 protein expression, causing a dominant negative reduction in I_Na_ density and consequently decreased conduction velocity [2,6]. The effect of activated PERK is not specific to Na_v_1.5. UPR activation also results in a reduction of *KCND3* that encodes the α subunit of K_v_4.3. K_v_4.3 conducts I_to_, which is the main contributor to the notch of phase 1 of the cardiac AP and is responsible for early repolarization. Reduced I_to_ can increase membrane resistance, causing shortening of the cardiac action potential duration and phase 2 reentry [13,63]. Blocking PERK prevents these ion channel downregulations [6].

**Figure 2.**
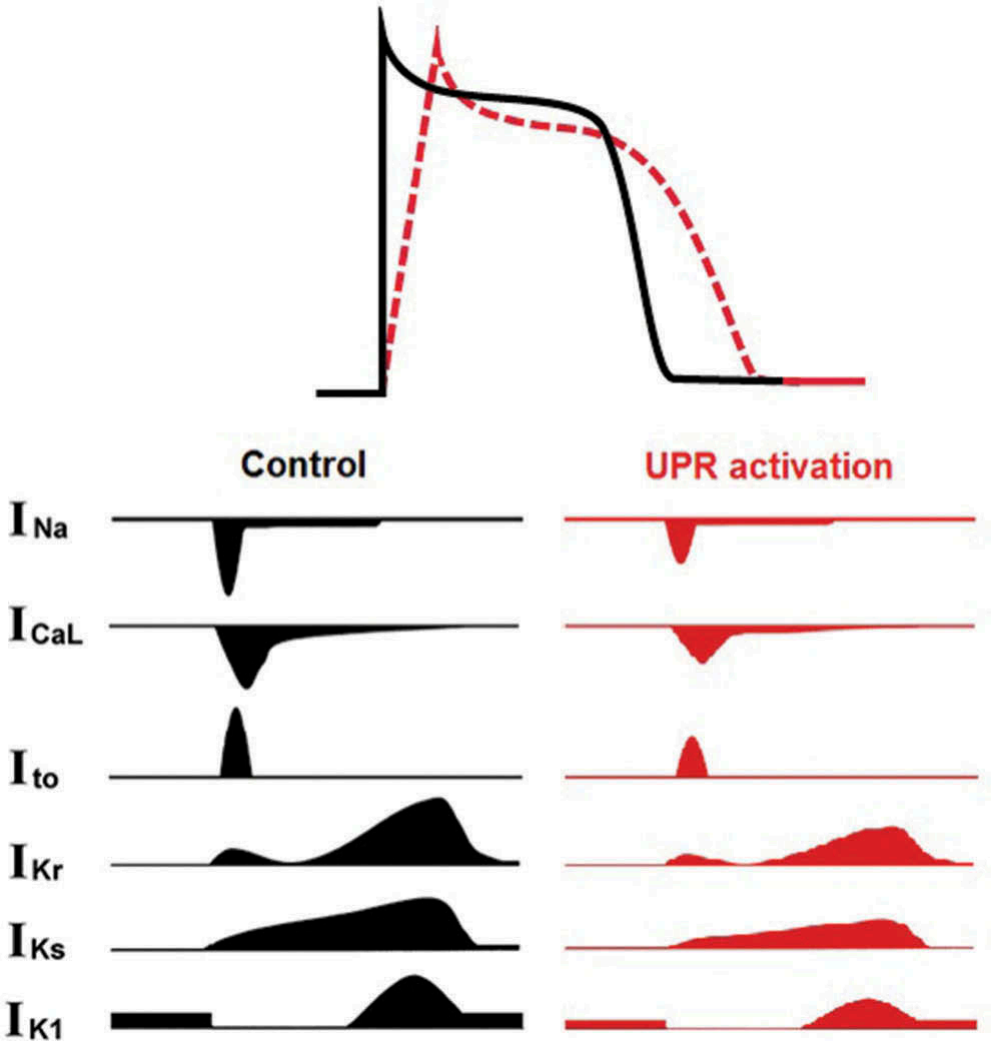
Tunicamycin-induced UPR activation alters the morphology of the action potential with prolonged action potential duration and decreased dV/dt_max_ of hiPSC-CMs, by decreasing all major cardiac ion channel currents.

**Figure 3.**
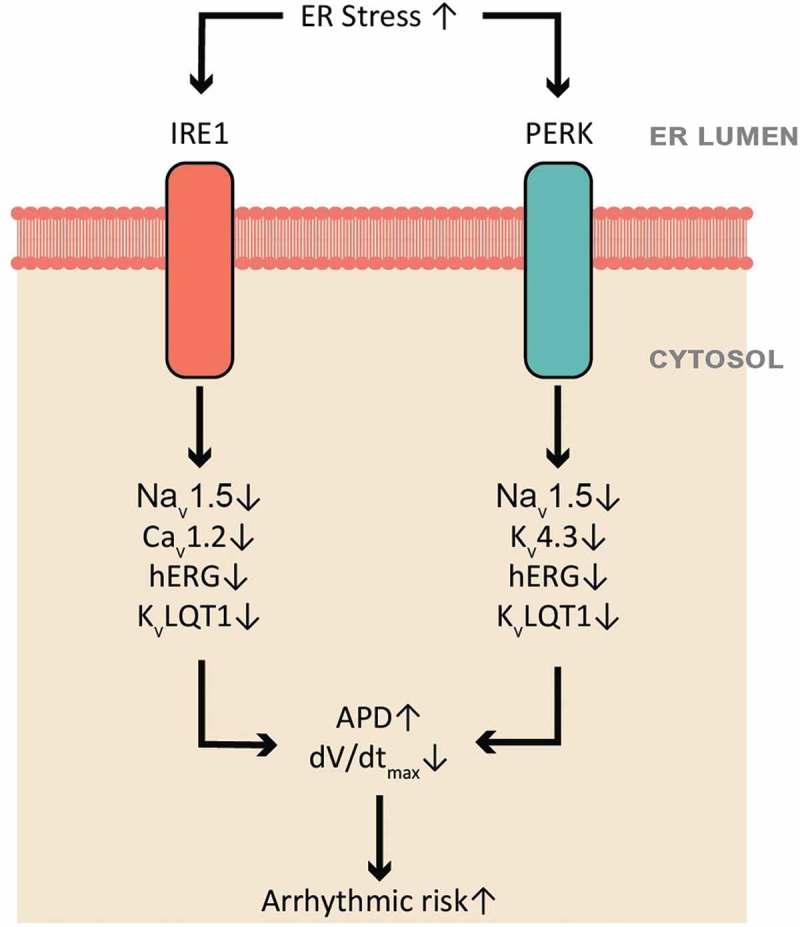
A summarized scheme of the UPR regulation on human cardiac ion channels. Activated UPR downregulates selective ion channels, leads to prolonged APD and reduced dV/dt_max_, which can contribute to electrical remodeling and arrhythmias. The PERK branch downregulates Na_v_1.5, K_v_4.3, hERG, and K_v_LQT1, while the IRE1 branch downregulates Na_v_1.5, Ca_v_1.2, hERG, and K_v_LQT1.

The UPR may contribute to downregulations of other ion channels in heart failure. Recently, our group reported downregulation of multiple cardiac ion channels by tunicamycin-induced UPR activation in human induced pluripotent stem cell-derived cardiomyocytes (hiPSC-CMs) (Figure 2) [62]. The downregulation of multiple cardiac ion channels corresponds to the APD prolongation and dV/dtmax reduction [62]. With the help of specific inhibitors of the PERK and IRE1 arm, we identified PERK-dependent regulation of Na_v_1.5, K_v_4.3, human rapid delayed rectifying K^+^ channel (hERG), and K_v_LQT1 but not Ca_v_1.2, and IRE1-dependent regulation of Na_v_1.5, Ca_v_1.2, hERG, and K_v_LQT1 but not of K_v_4.3 (Figure 3) [62]. Inhibition of either the PERK or IRE1 arm led to a shortened APD and recovered dV/dt_max_. This suggests that PERK- and IRE1-mediated channel downregulations are specific for a certain set of channels, and that some cross talk exists between the PERK and IRE1 arms of UPR. The determinants of the specificity on channel regulation are unknown currently. Some ion channel β subunits are also downregulated in cardiomyopathy. The extent to which these changes are the result of the UPR requires further investigation.

Activated UPR induces a positive shift of the V_1/2_ of I_Na_ steady state activation that can be reversed partially by PERK inhibition with GSK2606414 and completely by IRE1 inhibition with 4µ8C [62], suggesting that the UPR results in changes in post-translational modifications of Na_v_1.5 or possibly changes in associated channel subunits. It is noteworthy that inhibition of IRE1 under physiological condition downregulates Ca_v_1.2, hERG and K_v_LQT1 and prolongs the APD, indicating that certain IRE1 activity may be necessary to maintain these channels under physiological conditions. Inhibition of either PERK or IRE1 shows partial reversal of electrical remodeling, suggesting that the ATF6α arm may play a role in electrical remodeling or that there are overlapping effects of the UPR arms.

It is likely that some of the UPR effects are secondary to UPR-mediated changes that indirectly modify cardiac electrophysiology. For example, the activated UPR may affect ion channels or pumps that determine the resting membrane potentialf, thereby affecting the behavior of voltage-gated channels. Kir2.x, Na/K pump, Kir3.x (conducting I_K,ACh_) and Kir6.2 (conducting I_K,ATP_) in atria, and HCN channels (conducting I_f_) for nodal cells are important determinants of the maximum diastolic depolarization. Downregulations of some of these channels have been reported in cardiomyopathy [64–68]. The role of the UPR in these changes remains to be determined.

Oxidative stress induced by the UPR [50,69,70] can also modulate cardiac ion channels [7,71,72]. Other mechanisms of arrhythmia under ER stress include altered Ca^2+^ homeostasis. Under prolonged ER stress, Ca^2+^ release from the ER is increased [19], which can not only activate signaling pathways of cell apoptosis but also induce triggered activity that can cause cardiac arrhythmias. ER-dependent ion channel glycosylation is also altered with UPR activation. A recent study shows that only a fully glycosylated cardiac Na_v_1.5 is trafficked normally [73]. Our group observed altered Na_v_1.5 glycosylation under UPR activation, which was reversed by IRE1 inhibition (unpublished data). Therefore, altered glycosylation during ER stress may contribute to ion channel alterations and arrhythmic risk.

## Blocking UPR to raise ion channels as a treatment of arrhythmia

There is considerable literature to support the idea that UPR inhibition is beneficial in heart disease including ischemia/reperfusion [74], acute myocardial infarction [48,53,75,76], and chronic ischemic heart failure [47,57,77]. None of these studies looked at electrical remodeling, however. Our recent study shows that activated UPR downregulates multiple cardiac ion channels [6,62]. Therefore, blocking the UPR to reverse arrhythmogenic channel downregulation may be a new paradigm of maintaining ion channel activity to prevent arrhythmia. Electrical remodeling can be partially reversed by inhibition of either the PERK or IRE1 arm [62]. Chemical chaperones, such as 4-phenylbutyric acid (4-PBA) and taurine-conjugated ursodeoxycholic acid (TUDCA) have been reported to reduce the ER stress by preventing misfolded protein aggregation and by suppressing the elevation of UPR sensors and effectors in hypertension and obesity-induced cardiac hypertrophy [78,79]. They show significant effects on reduced cardiac damage, improved vascular function in hypertension, and alleviation of compromised fractional shortening and cardiomyocyte contractile [78,79]. Analogues of 4-PBA such as 2-POAA-OMe, 2-POAA-NO_2_, and 2-NOAA have been found to suppress the induction of Grp78 and CHOP and to inhibit the IRE1 and ATF6α pathways [80]. Therefore, TUDCA, 4-PBA and its analogues may be used to suppress UPR activation and reverse the downregulation of cardiac ion channels.

While blocking the UPR activation appears logical to prevent arrhythmias, this signaling cascade has shown to have protective and harmful effects in certain circumstances [23,81–83]. On one hand, homozygous knockout animal models of several UPR sensors and effectors are potentially harmful or even lethal (reviewed in [84]). On the other hand, inhibition of the PERK arm with apelin-13 protects the heart from ischemia-induced ER stress [85]. Mice with inducible cardiac-specific PERK knockout have been shown to have normal heart function and not suffer from diabetes [53]. Nevertheless, the mouse heart ejection fraction is slightly decreased in response to chronic transverse aortic constriction when compared to control mice [53]. Therefore, partial or temporary inhibition of the UPR sensors or organ-specific gene therapy may be the safer alternative. Moreover, timing of inhibition may matter. Further investigation and understanding of the UPR will help overcome these limitations and discover more precisely targeting agents or time-limited treatment approaches.

## Summary

**Figure 4.**
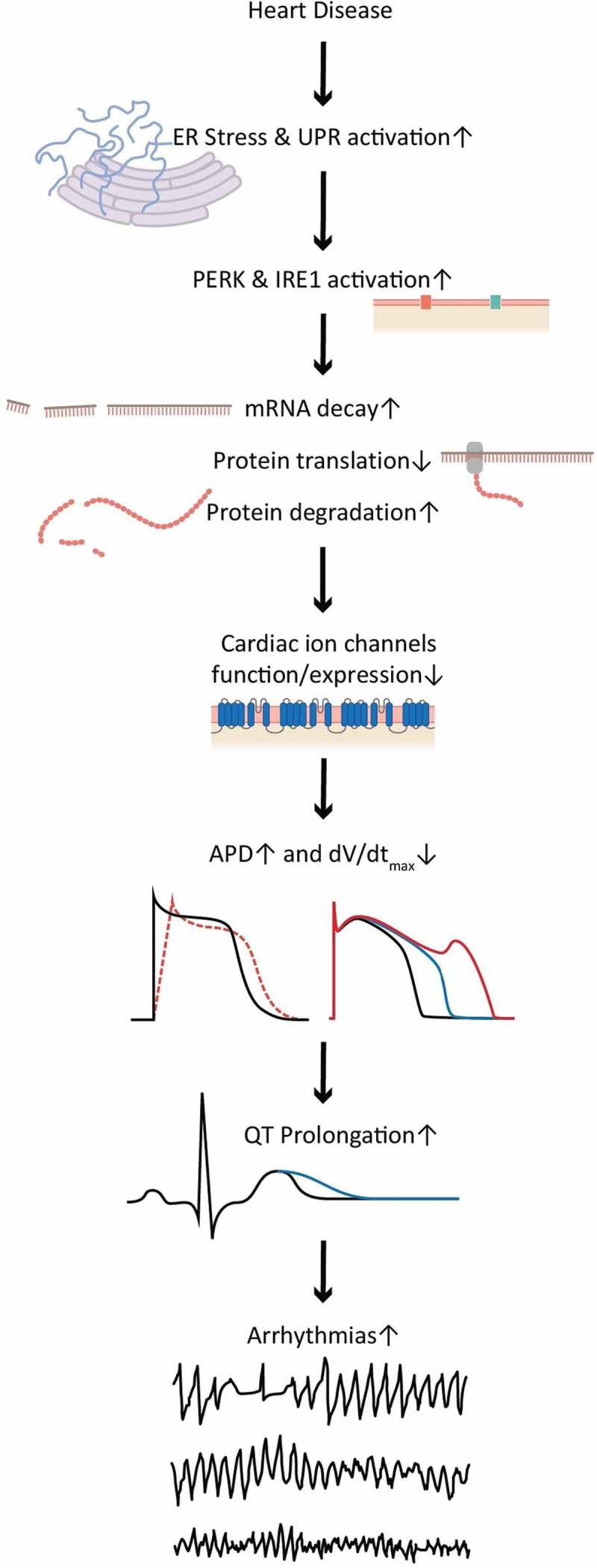
A summary of the UPR activation causing arrhythmias.

Cardiac injury results in UPR activation, which induces ion channel downregulations. These downregulations can explain some of the arrhythmic risk (Figure 4). Blocking UPR activation can raise ion channel levels and may represent a new and potentially fruitful antiarrhythmic paradigm.
